# Effect of soy lecithin on fatigue and menopausal symptoms in middle-aged women: a randomized, double-blind, placebo-controlled study

**DOI:** 10.1186/s12937-018-0314-5

**Published:** 2018-01-08

**Authors:** Asuka Hirose, Masakazu Terauchi, Yurika Osaka, Mihoko Akiyoshi, Kiyoko Kato, Naoyuki Miyasaka

**Affiliations:** 1 Department of Obstetrics and Gynecology, Tokyo Medical and Dental University, Yushima 1-5-45, Bunkyo, Tokyo, 113-8510 Japan; 2 Department of Women‘s Health, Tokyo Medical and Dental University, Yushima 1-5-45, Bunkyo, Tokyo, 113-8510 Japan

**Keywords:** Atherosclerosis, Cardiovascular risks, Fatigue, Lipid replacement therapy, Menopausal symptoms, Psychological symptoms, Soy

## Abstract

**Background:**

Lecithin is a complex mixture of phospholipids which compose lipid bilayer cell membranes. Lipid replacement therapy, or administration of phospholipids for the purpose of repairing the dmaged cell membranes, had been shown to alleviate fatigue. The present study aimed to investigate the effect of soy lecithin on fatigue in middle-aged women, as well as other menopausal symptoms and various health parameters.

**Methods:**

This randomized, double-blind, placebo-controlled study included 96 women aged 40 to 60 years who complained of fatigue. The participants were randomized to receive active tablets containing high-dose (1200 mg/day; *n* = 32) or low-dose (600 mg/day; *n* = 32) soy lecithin, or placebo (*n* = 32), for 8 weeks. The following parameters were evaluated: age, menopausal status, lifestyle factors, physical and psychological symptoms of menopause, subjective symptoms of insomnia and fatigue, body composition, cardiovascular parameters, and physical activities and objective sleep states obtained from actigraphy before and 4 and 8 weeks after treatment. Fatigue was evaluated using the Profile of Mood States (POMS)-brief, Menopausal Health-Related Quality of Life questionnaire, Chalder Fatigue Scale, and Brief Fatigue Inventory.

**Results:**

Eighty-nine women completed the study. There were no significant differences in the changes in Chalder Fatigue Scale score (placebo vs low-dose vs high-dose groups: −2.9 ± 1.1, −3.2 ± 1.1, and −3.5 ± 1.0; *P* = 0.79). On the other hand, the improvements were greater in the high-dose group compared with the placebo group concerning vigor scores in the POMS-brief (1.9 ± 0.7 vs 0.2 ± 0.6; *P* = 0.02), diastolic blood pressure (−4.1 ± 1.8 vs 1.2 ± 1.9; *P* = 0.05), and cardio-ankle vascular index (−0.4 ± 0.2 vs 0.07 ± 0.1; *P* = 0.03) after 8 weeks of treatment.

**Conclusions:**

High-dose (1200 mg/day) soy lecithin not only increases vigor, but also lowers the diastolic blood pressure and cardio-ankle vascular index in middle-aged women who present with fatigue.

**Trial registration:**

UMIN-CTR UMIN000017127.

## Background

Middle-aged women are bothered by various symptoms such as hot flashes, night sweats, anxiety, depression, insomnia, and fatigue. A previous study revealed that 86% of Japanese middle-aged women who visited our menopausal clinic felt tiredness more than once a week, and 49% felt it almost every day [1]. Another study revealed that 85% of Japanese and 82% of Australian middle-aged women felt fatigue, at least to some extent [2]. Compared to the patients attending a primary care clinic of whom 14% complained of fatigue [3], apparently more middle-aged women are bothered by the symptom.

Lecithin is a complex mixture of phospholipids found mainly in egg yolks, soy, and coleseeds. Phospholipids such as phosphatidyl choline, phosphatidyl ethanolamine, phosphatidyl inositol, phosphatidyl serine, and phosphatidyl glycerol compose the lipid bilayer of the cellular membrane structure. Membrane lipids are used for energy storage necessary for membrane biogenesis [4–6]. The matrix of cellular membranes is formed by polar lipids, which consist of a hydrophobic and hydrophilic portion and segregate the internal and external environment [6]. Furthermore, membrane lipids act as first and second messengers in signal transduction and molecular recognition pathways [4, 6], implying the variability of their biological roles. The administration of fresh phospholipids to replace damaged cell membranes and restore the structure and function of the cellular membrane is called lipid replacement therapy (LRT). LRT has been shown to improve fatigue, and is expected to be effective for the treatment of degenerative diseases, metabolic syndrome, diabetes, and cardiovascular diseases [4, 7]. However, previous studies have demonstrated the effects of LRT using a supplement containing not only phospholipids but also other active ingredients. To the best of our knowledge, this study is the first to examine the effects of soy lecithin on fatigue.

The present study aimed to investigate the effect of soy lecithin on menopausal symptoms including fatigue and other various health parameters in Japanese middle-aged women.

## Methods

### Study population

We performed a randomized, double-blind, placebo-controlled study from February to July 2015 at the Menopause Clinic of Tokyo Medical and Dental University. The study protocol was reviewed and approved by the Tokyo Medical and Dental University Review Board, and written informed consent was obtained from all the participants. The present study was conducted in accordance with the Declaration of Helsinki.

Ninety-six Japanese women aged 40 to 59 years who complained of fatigue participated in this study. Fatigue was evaluated using the item about fatigue in the Menopausal Health-Related Quality of Life (MHR-QOL) questionnaire. The participants were recruited through advertisements posted in our hospital and in the patients’ social network. Those who were receiving menopausal hormone therapy, herbal medicine, or psychotropic drugs were excluded. Collected data included age, menopausal status, lifestyle factors, physical and psychological symptoms of menopause, subjective symptoms of insomnia and fatigue, body composition, cardiovascular parameters, and physical activities and objective sleep states obtained from actigraphy. The data were evaluated before and 4 and 8 weeks after treatment. Regarding menopausal status, participants were classified as follows: premenopausal (regular menstrual cycles in the past 3 months), perimenopausal (a menstrual period within the past 12 months but a missed period or irregular cycles in the past 3 months), postmenopausal (no menstrual period in the past 12 months), or surgically induced menopause (hysterectomy).

### Randomization and intervention

The participants were randomized into 1 of 3 groups to receive active tablets containing high-dose (1200 mg/day; *n* = 32) or low-dose (600 mg/day; *n* = 32) soy lecithin, or placebo (n = 32) for 8 weeks. The high-dose and low-dose soy lecithin and placebo tablets, indistinguishable in shape, weight, and color, were manufactured and packaged by Kikkoman Corporation (Noda, Japan). The tablet contained phospholipids in the form of phosphatidylcholine, 24%; phosphatidylethanolamine, 20%; and phosphatidylinositol, 12%. The women were instructed to take six tablets per day after breakfast. Medication adherence was evaluated by collecting the packages of the supplements from participants. The participants were sequentially numbered, and received supplement packages with the corresponding number. The content of each package, namely high-dose or low-dose soy lecithin, or placebo, was known only by the manufacturers. Thus, allocation was concealed from both the participants and the investigators. Safety was assessed by patient-reported treatment-emergent adverse events.

### Menopausal symptoms

Menopausal symptoms were evaluated using the Menopausal Health-Related Quality of Life (MHR-QOL) questionnaire, Hospital Anxiety and Depression Scale (HADS), and Athens Insomnia Scale (AIS). The MHR-QOL questionnaire, developed and validated at our clinic [8, 9], is a modification of the Women’s Health Questionnaire [10, 11], and contains 38 items scored on a 4-point or binary scale covering four major domains (physical symptoms, psychological symptoms, life satisfaction, and social involvement) of a woman’s health during menopause. The HADS [12] is a reliable instrument for screening clinically significant anxiety and depression in women visiting a general medicine clinic. The translated Japanese version of the HADS [13] was used in the present study. The AIS was developed as a brief and easy-to-administer self-assessment questionnaire for determining the severity of insomnia defined according to the International Classification of Diseases 10th Revision. The internal consistency and test-retest reliability of the AIS have been confirmed previously [14]. Detailed information about the MHR-QOL questionnaire, HADS, and AIS is provided elsewhere [8].

### Fatigue and mood states

Fatigue and mood states were evaluated by the Profile of Mood States (POMS)-brief, Chalder Fatigue Scale (CFS), and Brief Fatigue Inventory (BFI). The POMS is a psychological rating scale used to assess feelings and mood states. The original version of the POMS consists of 65 items, while the POMS-brief, a shortened version, includes 30 items covering six major domains (tension, depression, anger, vigor, fatigue, and confusion). Because the latter is easier to complete and its validity has been demonstrated [15], it is now more widely used than the original form. The POMS-brief was translated into Japanese, and its reliability and validity were confirmed previously [16]. The CFS is a self-administered questionnaire with 14 items which evaluates fatigue-related symptoms by using a 4-point Likert scale for the extent or frequency of the symptoms [17]. Although originally developed to measure fatigue symptoms in clinical settings, the scale is now more widely used [18]. The BFI, a self-rating assessment composed of 9 items using a numerical scale of 0 to 10, was developed for the rapid assessment of fatigue severity in cancer patients [19]. It was translated into Japanese, and its reliability and validity were confirmed previously [20].

### Body composition and cardiovascular parameters

Height, weight, body mass index, and body composition including fat mass, and fat free mass, was assessed using a body composition analyzer (MC190-EM; Tanita, Tokyo, Japan). Cardiovascular parameters, including systolic and diastolic blood pressure, heart rate, and the cardio-ankle vascular index (CAVI), were measured using a vascular screening system (VS-1000; Fukuda Denshi Co., Tokyo, Japan).

### Physical activities and objective sleep states

We investigated physical activities and objective sleep states using the actigraphy system that is loaded with a miniature triaxial acceleration sensor (Wrist-worn Accelerometers; Hitachi Ltd., Tokyo, Japan). It measured motion with accelerometers and sleep–wake identification was automatically performed by using the Cole–Kripke algorithm. The outcome measures included zero-crossing (ZC) and metabolic equivalents (METs) during the awake and sleep phase, total sleep time, day-to-day variation of sleep time, episodes of nocturnal awakening, sleep latency, and sleep efficiency. ZC is the rate of signal changes from the triaxial acceleration sensor, representing the frequency of wrist motion. METs are calculated as the ratio of metabolic rate relative to that in resting, representing the intensity of exercise. We define sleep latency as the time from time in bed to the start of identification of sleep. Sleep efficiency is calculated as pure sleep time (time in bed minus awake time) divided by time in bed. Participants were requested to wear a wrist actigraph device for 72 h on their non-dominant arms except during bathing time. The analysis of the actigraphy data was performed at ‘blinded for peer review’.

### Statistical analyses

The baseline characteristics of the participants who completed the 8-week treatment were compared using one-way analysis of variance, χ2 test, and Kruskal-Wallis test. Then, the changes from baseline to 8 weeks of treatment in all of the collected data were evaluated using Mann-Whitney test and unpaired t-test. All statistical analyses were performed with GraphPad Prism version 5.02 (GraphPad Software Incorporated, CA, USA). A *P*-value <0.05 was considered statistically significant.

## Results

**Fig. 1:**
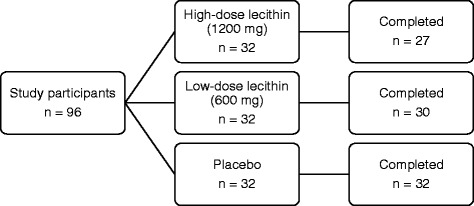
Participant disposition

A total of 96 middle-aged women were enrolled in the study and were randomized to the high-dose (*n* = 32), low-dose (n = 32), or placebo groups (*n* = 32); of these, 89 (94%) completed the 8-week treatment period (Fig. 1). During the study period, seven women dropped out of the study. Five of them did not continue to take the supplements, and two did not have an examination at our clinic. The baseline characteristics of the participants who completed the 8-week treatment are shown in Table 1. The group mean age ranged from 49 to 51 years. The proportion of women who were premenopausal, perimenopausal, postmenopausal, and had surgically induced menopause ranged from 22 to 52%, 7 to 22%, 41 to 53%, and 0 to 7%, respectively. The weight, body mass index, and body fat mass were significantly higher in the high-dose group than the other groups (*P* = 0.018, 0.012, and 0.008, respectively).

**Table 1:** Background characteristics of the participants

	Placebo (*n* = 32)	Low-dose lecithin (*n* = 30)	High-dose lecithin (*n* = 27)	*P* value
Age, y	51.2 ± 5.2	50.3 ± 5.8	49.3 ± 4.8	0.423^a^
Menopausal status, n (%)				
Premenopausal	7 (21.9)	10 (33.3)	14 (51.9)	0.222^b^
Perimenopausal	7 (21.9)	5 (16.7)	2 (7.4)	
Postmenopausal	17 (53.1)	13 (43.3)	11 (40.7)	
Surgically induced menopause	1 (3.1)	2 (6.7)	0 (0)	
Questionnaire				
MHR-QOL physical symptom score	21.2 ± 3.5	21.9 ± 2.9	20.6 ± 3.2	0.406^c^
MHR-QOL psychological symptom score	27.1 ± 8.0	29.7 ± 6.7	30.0 ± 5.5	0.328^c^
HADS-anxiety subscale score	5.8 ± 2.9	5.3 ± 3.3	4.9 ± 2.7	0.549^c^
HADS-depression subscale score	5.5 ± 3.7	4.8 ± 3.7	4.7 ± 3.1	0.679^c^
AIS score	5.0 ± 3.0	4.0 ± 2.9	4.1 ± 2.6	0.443^c^
Chalder Fatigue Scale	18.7 ± 8.3	17.1 ± 5.3	17.6 ± 6.6	0.628^c^
Brief Fatigue Inventory	2.7 ± 1.9	3.0 ± 1.7	3.0 ± 2.0	0.575^c^
POMS-tension	6.1 ± 4.9	5.5 ± 4.3	4.3 ± 3.1	0.461^c^
POMS-depression	3.9 ± 3.9	2.5 ± 3.1	2.5 ± 2.6	0.217^c^
POMS-anger	5.1 ± 4.2	3.7 ± 3.1	2.8 ± 2.5	0.104^c^
POMS-vigor	7.7 ± 4.1	7.2 ± 3.6	6.1 ± 3.5	0.179^c^
POMS-fatigue	6.8 ± 4.4	7.8 ± 4.3	6.4 ± 3.6	0.727^c^
POMS-confusion	5.0 ± 2.8	4.8 ± 2.4	3.8 ± 2.2	0.323^c^
Body composition				
Height, cm	157.6 ± 4.6	158.4 ± 5.1	158.0 ± 3.8	0.817^a^
Weight, kg	52.8 ± 7.5	52.6 ± 6.0	58.3 ± 11.1	0.018^a^
Body mass index, kg/cm^2^	21.2 ± 2.8	21.0 ± 2.5	23.3 ± 4.0	0.012^a^
Body fat mass, kg	14.0 ± 5.1	13.7 ± 4.4	18.5 ± 8.5	0.008^a^
Fat free mass, kg	36.5 ± 2.8	36.6 ± 2.2	37.6 ± 2.8	0.222^a^
Waist:hip ratio, %	80.6 ± 6.1	81.7 ± 4.8	83.4 ± 6.2	0.191^a^
Body temperature, °C	36.3 ± 0.33	36.4 ± 0.42	36.3 ± 0.41	0.701^a^
Cardiovascular parameters				
Systolic blood pressure, mmHg	122.7 ± 15.7	119.3 ± 16.1	119.9 ± 13.6	0.668^a^
Diastolic blood pressure, mmHg	69.2 ± 12.1	67.8 ± 13.6	68.7 ± 13.6	0.919^a^
Heart rate, beats/min	77.2 ± 8.0	76.9 ± 12.7	80.1 ± 12.4	0.504^a^
Cardio-ankle vascular index	7.19 ± 0.79	7.24 ± 0.79	7.14 ± 0.78	0.967^a^
Actigraphy				
Active ZC (/min)	156.6 ± 26.2	148.0 ± 31.8	149.0 ± 34.0	0.506^a^
Sleep ZC (/min)	4.3 ± 1.1	4.3 ± 1.8	4.7 ± 1.7	0.606^a^
Active METs	1.6 ± 0.15	1.5 ± 0.14	1.6 ± 0.21	0.674^a^
Sleep METs	1.1 ± 0.015	1.1 ± 0.015	1.1 ± 0.019	0.580^a^
Sleep time (min/day)	372.1 ± 70.6	371.4 ± 60,6	367.8 ± 54.8	0.687^a^
Sleep efficiency (%)	97.2 ± 1.4	97.4 ± 1.7	97.5 ± 1.8	0.719^a^
Episodes of nocturnal awakening (>1 min) (/day)	1.7 ± 1.2	1.8 ± 1.4	1.8 ± 1.5	0.676^a^
Lifestyle factors, %				
Exercising regularly	50.0	46.7	51.9	0.923^b^
Smoking	6.25	10.0	7.4	0.856^b^
Drinking alcohol (daily / by chance / not at all)	15.6 / 68.8 / 15.6	20.0 / 63.3 / 16.7	29.6 / 59.3 / 11.1	0.749^b^

Values are presented as mean ± SD unless otherwise indicated

*AIS* Athens Insomnia Scale, *CAVI* cardio-ankle vascular index, *HADS* Hospital Anxiety and Depression Scale, *METs* metabolic equivalents, *MHR-QOL* Menopausal Health-Related Quality of Life, *POMS* Profile of Mood States, *ZC*, zero-crossing

^a^One-way analysis of variance

^b^Chi-square test

^c^Kruskal-Wallis test

**Fig. 2:**
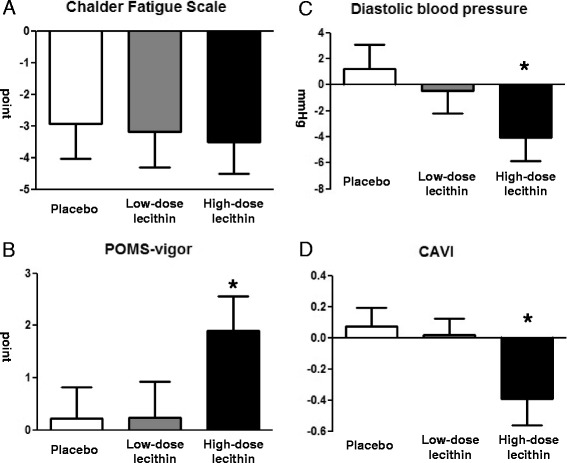
The changes from baseline after 8 weeks of intervention in: (**a**) the Chalder Fatigue Scale scores; (**b**) the Profile of Mood States (POMS)-vigor scores; (**c**) diastolic blood pressure; and (**d**) cardio-ankle vascular index (CAVI). Data are presented as means and standard errors. *P < 0.05 vs placebo, Mann-Whitney test or unpaired t-test

The mean ± SEM CFS scores significantly decreased after 8 weeks of treatment in all the groups, and there were no significant differences among the groups (placebo vs low-dose vs high-dose groups: −2.9 ± 1.1, −3.2 ± 1.1, and −3.5 ± 1.0, respectively; *P* = 0.79, Kruskal-Wallis test, Fig. 2a). After 8 weeks of treatment, the mean ± SEM POMS-vigor score was significantly higher in the high-dose group than the placebo group (1.9 ± 0.7 vs 0.2 ± 0.6, respectively; *P* = 0.02, Mann-Whitney test, Fig. 2b). The mean ± SEM diastolic blood pressure (−4.1 ± 1.8 vs 1.2 ± 1.9; *P* = 0.05, unpaired t-test, Fig. 2c) and CAVI (−0.4 ± 0.2 vs 0.07 ± 0.1; *P* = 0.03, unpaired t-test, Fig. 2d) significantly decreased after 8 weeks of treatment in the high-dose group compared with the placebo group. Neither the body composition parameters nor the actigraphic parameters changed significantly in any of the groups (data not shown).

During the whole study period, no treatment-emergent adverse event was reported by the participants, even though laboratory tests were not routinely performed.

## Discussion

In this randomized, double-blind, placebo-controlled study, the effect of soy lecithin on menopausal symptoms including fatigue and other various health parameters in Japanese middle-aged women was investigated. The results showed that high-dose (1200 mg/day) soy lecithin not only increases vigor, but also lowers the diastolic blood pressure and CAVI in Japanese middle-aged women who complained of fatigue.

LRT is a concept proposed by Nicolson et al. [4]. The mitochondrion is the site where adenosine triphosphate (ATP) is synthesized aerobically, and the inner and outer membrane of the organelle are composed by phospholipids. Reactive oxygen species (ROS) are generated in the process of uncoupling the electron transport chain from ATP generation, with the inner mitochondrial membrane being the most important source of ROS [4, 21]. When mitochondrial membranes are damaged by ROS, energy production by the organelle is decreased, which could lead to fatigue, as well as aging and various diseases. In previous animal [22] and clinical studies [23], LRT was demonstrated to improve mitochondrial function assessed by transport and reduction of the dye Rhodamine-123, mitochondrial membrane potentials assessed by flow cytometry, and mitochondrial DNA deletions [24].

Lecithin is particularly abundant in egg yolks, soybeans, organ and lean meats, and fish. The total phospholipids contained in 100 g of egg yolk and soybeans are 31.8 g and 20.8 g, respectively [25], and the normal dietary intake of phospholipids is estimated as 2–8 g per day [26]. Most previous studies [4, 7, 26] have used the dosage of 1 to 3 g per day of oral supplementation of lecithin, exhibiting its effects for inflammatory diseases, hyperlipidemia, cardiovascular diseases, cognitive impairment, and fatiguing illnesses. Oral administration of phospholipids has been shown to be generally safe [4]. For example, in phase I and II clinical trials in patients with cardiovascular diseases, over 5 g of soy phospholipids were administered with no apparent toxicity [27]. Referring to older studies, a daily dosage of as much as 54 g has been administered without any apparent adverse effects [28]. Regarding the duration of the treatment, most of the previous studies investigating the effects of dietary LRT supplement on fatigue examined for 8 weeks, meanwhile some examined for 1 or 12 weeks [4].

In this study, the effect of soy lecithin on fatigue and other factors was investigated, and our findings showed that lecithin promotes vigor assessed by the POMS-brief questionnaire. Previous studies have demonstrated the effect of phospholipids on cancer-associated fatigue and adverse chemotherapy effects, such as nausea, diarrhea, constipation, and fatigue [29, 30], using NT Factor® (Nutritional Therapeutics, Inc., Hauppauge, NY.), an oral supplement containing phospholipids, glycolipids, and other membrane lipids [23]. Feelings of fatigue and loss of vigor are similar and the difference is ambiguous, and our result can be understood in the same context. In this study, significant differences in the fatigue scores among the groups could not be shown. The reason for this may be the placebo effect because the fatigue scores were improved in all groups, and it was explained to the participants that lecithin might improve fatigue. Other reasons why the improvement of fatigue was not different among the groups might be that the participants were relatively healthy and the baseline fatigue scores were not high enough, or that some portion of the fatigue experienced by the participants was derived from pathological conditions, such as infection, inflammation, and sleep disorders.

The present study also revealed that lecithin decreases the diastolic blood pressure and CAVI. The risk of cardiovascular diseases (CVDs), including central obesity, hypertension, dyslipidemia, and diabetes, increases after menopause [31–34], partly as a result of diminished estrogen production [35]. CVD is rare in young women, but it is the second leading cause of death among the 45–64 year age group in the United States after cancer, and it has become the leading cause after the age of 65 years [36]. Especially in women, the treatment of arterial hypertension and diabetes is considered important for the prevention of CVD [37]. Several studies demonstrated that lecithin lowered total cholesterol in patients with primary hyperlipidemia [38, 39], and a mini-pig study showed that LRT reduced atherosclerotic plaques in the aortas and heart valves [40]. There is a possibility that lecithin lowers cardiovascular risks, although the mechanisms are yet to be elucidated. This is the first study showing the effects of lecithin on the diastolic blood pressure and CAVI. Further study is warranted to corroborate the findings and also to examine the effect on systolic blood pressure. In the present study, there was statistically significant differences in the baseline characteristics among the study groups, that is weight, body mass index, and body fat mass were significantly higher in the high-dose group. On the other hand, there was no significant difference in the POMS-vigor score, diastolic blood pressure, and CAVI that changed significantly after the 8-week treatment, therefore we consider that the differences in the baseline characteristics would have a small influence on the results of the present study.

The present study has some limitations. First, the sample size was relatively small and the study period was as short as 8 weeks, and all the participants were relatively healthy women. Second, mitochondrial function was not evaluated, and the results could not be directly explained by the functional improvement of the mitochondria. Finally, we set the full dose as 1200 mg in this study, which might not be sufficient. Further studies with longer duration enrolling more patients with severe fatigue, hypertension, or established atherosclerosis are warranted in order to corroborate our findings concerning the effects of soy lecithin on vigor and cardiovascular risks. Higher doses of lecithin and simultaneous assessment of the mitochondrial function may be needed to evaluate the effects of the compound.

## Conclusions

High-dose (1200 mg/day) soy lecithin not only increases vigor, but also lowers the diastolic blood pressure and CAVI in middle-aged women who present with fatigue. Further studies with longer duration enrolling more patients with severe fatigue are warranted in order to corroborate our findings.

## Abbreviations

AIS: Athens insomnia scale
ATP: Adenosine triphosphate
BFI: Brief fatigue inventory
CAVI: Cardio-ankle vascular index
CFS: Chalder fatigue scale
CVD: Cardiovascular disease
HADS: Hospital anxiety and depression scale
LRT: Lipid replacement therapy
MET: Metabolic equivalent
MHR-QOL: Menopausal health-related quality of life
POMS: Profile of mood states
ROS: Reactive oxygen species
ZC: Zero-crossing
